# Anti-diabetic effect of anthocyanin cyanidin-3-O-glucoside: data from insulin resistant hepatocyte and diabetic mouse

**DOI:** 10.1038/s41387-024-00265-7

**Published:** 2024-03-01

**Authors:** Xiang Ye, Wen Chen, Xu-Fan Huang, Fu-Jie Yan, Shui-Guang Deng, Xiao-Dong Zheng, Peng-Fei Shan

**Affiliations:** 1 Department of Endocrinology and Metabolism, The Second Affiliated Hospital of Zhejiang University School of Medicine, 310058 Hangzhou, China; 2 College of Biosystems Engineering and Food Science, Zhejiang University, 310058 Hangzhou, China; 3 Advanced Computing and System Laboratory, College of Computer Science and Technology, Zhejiang University, 310058 Hangzhou, China; 4 Innovation Centre for Information, Binjiang Institute of Zhejiang University, 310058 Hangzhou, China

**Keywords:** Diabetes, Organelles

## Abstract

**Background:**

Anthocyanins are a group of natural products widely found in plants. They have been found to alleviate the disorders of glucose metabolism in type 2 diabetes mellitus (T2DM), while the underlying mechanisms remain unclear.

**Methods:**

HepG2 and L02 cells were incubated with 0.2 mM PA and 30 mM glucose for 24 h to induce IR, and cells treated with 5 mM glucose were used as the control. C57BL/6 J male mice and db/db male mice were fed with a chow diet and gavaged with pure water or cyanidin-3-O-glucoside (C3G) solution (150 mg/kg/day) for 6 weeks.

**Results:**

In this study, the anthocyanin C3G, extracted from red bayberry, was found to alleviate disorders of glucose metabolism, which resulted in increased insulin sensitivity in hepatocytes, and achieved by enhancing the glucose consumption as well as glycogen synthesis in insulin resistance (IR) hepatpcytes. Subsequently, the expression of key proteins involved in IR was detected by western blotting analysis. Protein tyrosine phosphatase-1B (PTP1B), a negative regulator of insulin signaling, could reduce cellular sensitivity to insulin by inhibiting the phosphorylation of insulin receptor substrate-2 (IRS-2). Results of this study showed that C3G inhibited the increase in PTP1B after high glucose and palmitic acid treatment. And this inhibition was accompanied by increased phosphorylation of IRS proteins. Furthermore, the effect of C3G on improving IR in vivo was validated by using a diabetic db/db mouse model.

**Conclusion:**

These findings demonstrated that C3G could alleviate IR in vitro and in vivo to increase insulin sensitivity, which may offer a new insight for regulating glucose metabolism during T2DM by using the natural dietary bioactive components.

**Figure:**
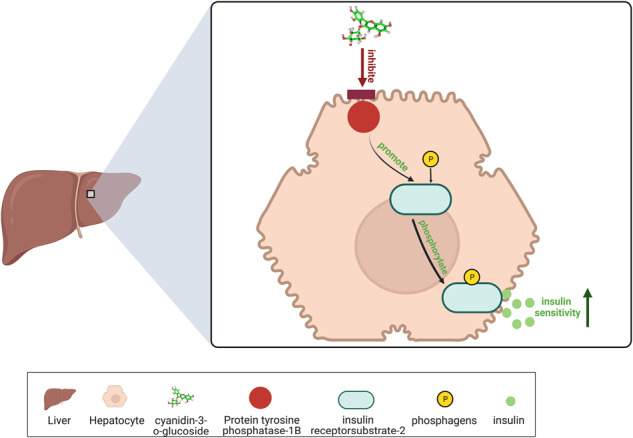
C3G promotes the phosphorylation of IRS-2 proteins by suppressing the expression of PTP1B, and then enhances the sensitivity of hepatocyte to insulin.

## Introduction

Diabetes mellitus characterized by chronic hyperglycemia, is one of the most prevalent chronic metabolic disease worldwide. Of these, type 2 diabetes mellitus (T2DM), which represents more than 90% of people with diabetes, is caused by the decrease in insulin sensitivity or insufficient insulin to overcome insulin resistance (IR) [1]. Therefore, the prevention and amelioration of T2DM can be achieved by increasing the insulin sensitivity of cells and tissues to improve the disorders of glucose metabolism.

Insulin exerts its effects by binding to receptors on the surface of insulin-sensitive tissues [2]. The insulin receptor is activated by insulin, and subsequently phosphorylates intracellular protein substrates, such as insulin receptor substrate-2 (IRS-2). Phosphorylation of IRS-2 induces activation of downstream proteins to participate in various biological processes, including the enhancement of cellular sensitivity to insulin [3]. In addition, PTP1B is considered as a negative regulator of insulin signaling [4], and one of the important mechanisms involved is the inhibition of IRS-2 phosphorylation.

At present, the treatment of T2DM has shifted from a primary reliance on multiple chemical drugs to natural products with fewer side effects. Although the chemical drugs could control the symptoms of T2DM to a certain extent, the accompanying side effects are difficult to avoid. Hence, natural products have received increasing attention due to their abundant sources, non-toxic advantages. Some studies have also shown that the intake of highly active natural products can improve the effectiveness of T2DM treatment and even reduce the amount of drugs taken [5–7].

Anthocyanins, a group of water-soluble natural compounds, are widely found in plants with dark pigmentation. Numerous scientific studies have proved the health benefits of anthocyanins from different plants, including anti-inflammatory, antioxidant, anti-tumor, anti-diabetic, anti-obesity, and anti-cardiovascular disease effects [8–10]. Yan et al. found that mulberry anthocyanin extract can reverse IR and enhance glucose metabolism in HepG2 cells by regulating the phosphatidylinositol 3 kinase/protein kinase B (PI3K/AKT) signaling pathway [11]. Another study has demonstrated that purple corn anthocyanins are beneficial in ameliorating tumor necrosis factor-α induced IR by activating insulin signaling and enhancing glucose transporter-4 (GLUT4) translocation in 3T3-L1 adipocytes [12]. Furthermore, a recent study has reported that aronia melanocarpa anthocyanin extracts (AMAE) could increase glucose uptake by modulating GLUT4 and up-regulating the p-GSK-3β(Ser9)/ GSK-3β ratio to strengthen glycogen synthesis in C2C12 and HepG2 cells with IR induced by palmitic acid (PA). Moreover, researchers have identified suppressor of cytokine signaling 3 as a key target gene involved in the process of enhancing insulin sensitivity and glycogen synthesis by AMAE [13]. These studies have provided preliminary evidence for the potential beneficial effects of anthocyanins on T2DM through their impacts in IR.

Anthocyanins are abundant in fresh berries, and red bayberry is particularly rich in anthocyanins. Red bayberry with powerful antioxidant capacity is used as a traditional Chinese medicine [14]. This study will also investigate the underlying molecular mechanisms, employing a combination of in vitro and in vivo experiments will be performed to investigate the role of anthocyanin cyanidin-3-O-glucoside (C3G) extracted from red bayberry in regulating glucose metabolism by alleviating IR status.

## Materials and methods

### Materials and reagents

Liver cell lines HepG2 and L02 were obtained from National Collection of Authenticated Cell Cultures (Shanghai, China). The glucose detection kit was obtained from RSbio (Shanghai, China). The glycogen stain kit was purchased from Jiancheng (Nanjing, China). Fluorescent probe of 2-NBDG was purchased from Dojindo (Shanghai, China). Recombinant human insulin was purchased from CoSin (Beijing, China). Pure C3G sample (No. SC8740) was purchased from Solarbio (Beijing, China). The primary antibodies used were PTP1B (Abcam, ab244207, 1:1000), anti-p-IRS-2 (Bioss, bs-0173R-1, 1: 250) and GAPDH (Abcam, ab8245, 1:10000). The secondary antibodies used were goat anti-rabbit (Sangon Biotech, D111018, 1:1000). The BCA kit, ECL kit, MTT kit and RIPA lysis buffer were purchased from Beyotime (Shanghai, China).

### Extraction and identification of anthocyanins from red bayberry

Anthocyanins in the fresh red bayberry fruit were extracted and identified according to the previously reported methods by our team [15]. For extraction, fresh red bayberry fruits were subjected to ultrasonic extraction in a 4-fold volume of anhydrous ethanol for 90 min. After sonicating, the mixture underwent filtration through a fine-grade mesh to discard the solid debris, and then the filtrate was evaporated at 49 °C using a rotary evaporator to remove the ethanol solvent. Subsequently, phase separation was performed by adding an equal volume of ethyl acetate to the concentrated extract and mixing. This mixture was allowed to stand for 10 min, and this process was repeated until a clear organic phase was achieved, after which the aqueous phase was collected for further purification. The aqueous phase was then loaded onto a D-101 macroporous resin column pre-equilibrated, and eluted with a solution of 1% formic acid in 80% methanol at a flow rate of 2 mL/min. The anthocyanin-rich fractions were concentrated under reduced pressure, and lyophilized using a vacuum freeze dryer operating at specified conditions to yield the dry anthocyanin extracts. Finally, the extracts were stored at −80 °C in an ultra-low temperature freezer until further use.

For identification of anthocyanins in the extracts, an ultra-high performance liquid chromatography (UPLC) system (Thermo UltiMate 3000, Thermo Fisher Scientific) was used. Chromatographic separation was achieved using a binary solvent system consisting of 1% formic acid in water (solvent A) and a mixture of acetonitrile with 0.1% formic acid aqueous solution (1:1, v/v; solvent B). Elution was performed following a linear gradient program where the concentration of solvent B was varied as follows: 10% to 38% over the first 40 min, held at 38% until 60 min, then increased to 48% by 60 min and further to 100% by 70 min, maintained at 100% for 5 min before returning to the original 10% composition over the next 5 min. The column was then equilibrated under initial conditions for 5 more min, resulting in a total run time of 80 min. The flow rate was maintained at 0.3 mL/min and the detection of anthocyanins was carried out at a wavelength of 520 nm.

### Cell culture and treatments

After reaching 80% confluence, HepG2 and L02 cells were incubated with 0.2 mM PA and 30 mM glucose for 24 h to induce IR [16], and cells treated with 5 mM glucose were used as the control (Con group). Then, IR hepatocytes were incubated with various concentrations (0, 40, or 80 μg/mL) of C3G for another 24 h.

### Cell viability assay

After treating with different concentrations of C3G for 24 h, HepG2 and L02 cells were treated with 100 μL of MTT with the final concentration of 0.5 mg/mL per well, and incubated at 37 °C for 4 h. Then, 150 µL of dimethyl sulfoxide was added to per well, shaking the plates until the formazan was completely dissolved. Finally, the absorbance value at 490 nm was measured by a microplate spectrophotometer.

### Glucose consumption and insulin sensitivity assays

HepG2 and L02 cells were seeded into a 96-well plate, the cell culture medium was collected after treatment, the glucose content of each well was measured with a commercial glucose detection kit. Next, the glucose consumption was calculated by the glucose content of the blank group minus that of the treated groups, and the actual glucose consumption was adjusted using cell viability measured by MTT analysis.

Insulin sensitivity index (%) = (glucose consumption in C3G group with insulin - glucose consumption in C3G group without insulin)/(glucose consumption in control group with insulin - glucose consumption in control group without insulin) × 100%.

### Glucose uptake assay

HepG2 and L02 cells were seeded into a 24-well plate. Then, 0.1 mM 2-NBDG dye was added for 30 min incubation at 37 °C after treatment, cells were washed with PBS for three times. Finally, images were obtained by a fluorescence microscope and the fluorescence intensity was calculated using the ipwin 32 software.

### Glycogen synthesis and glycogen staining assays

To determine the glycogen synthesis, different concentrations of glucose solutions were prepared, and a glucose standard curve was established with glucose concentration as the horizontal axis and absorbance value as the vertical axis. Hepatocytes were collected into a centrifuge tube and centrifuged for 5 min at 4 °C and 4000 × *g*, the supernatant was discarded and dried naturally. The cell mass was obtained by subtracting the mass of centrifuge tube from the total mass. 0.5 mL of 30% KOH solution was added to each sample and shaken well before heating for 30 min using a boiling-water bath. Each sample was cooled sufficiently before adding 1.5 mL of ethanol and allowing it to stand for 30 min. The sample was then centrifuged at 12,000 × *g* for 15 min at 4 °C, and the supernatant was discarded. Immediately, the precipitate was collected, dissolved in 0.5 mL dd-H2O, and then mixed with 1 mL of 0.2% anthrone solution. Heated in a water bath at 100 °C for 20 min, and the optical density (OD) value was measured at 620 nm. The glucose concentration of each sample was calculated using the glucose standard curve formula and divided by the corresponding cell mass to obtain the glycogen content per unit mass of cells.

For the glycogen staining assay, after treatment with different concentrations of C3G, the medium was removed and washed three times with PBS. A glycogen staining kit was used to measure the glucose content of cells, and images were obtained with a light microscope.

### RNA isolation, reverse transcription, and qRT-PCR analysis

An RNA miniprep kit was used to isolate total RNA from cells, and the concentration and purity were determined by a nucleic acid and protein detector (Nanodrop, Thermo). Then reverse transcription was performed with an RT kit to obtain the corresponding cDNA. The qRT-PCR was performed with cDNA (1:20, v:v) on an Applied Biosystems Step One RT-PCR system by using the TB Green kit (Takara, Japan). Table 1 shows the primer sequences of target genes.

**Table 1:** Primer sequences for the qRT-PCR assay.

Name	Forward primer (5’-3’)	Reverse primer (5’-3’)
PTP1B	GCAGATCGACAAGTCCGGG	GCCACTCTACATGGGAAGTCC
GAPDH	GGTGAAGGTCGGAGTCAACG	CAAAGTTGTCATGGATGHACC

The primer sequences of PTP1B and GAPDH for the qRT-PCR assay.

### Western blotting analysis

Proteins were extracted from treated cells by using a cell lysis buffer kit, and the total protein concentration was measured using a BCA kit. PTP1B, p-IRS-2 and GAPDH from the total protein were separated by SDS-PAGE (12% gel). The targeted proteins were then transferred to a polyvinylidene difluoride (PVDF) membrane, and incubated with blocking solution containing 10% skim milk (w/v) for 1 h at room temperature, followed by overnight incubation with different primary antibodies at 4 °C. Subsequently, PVDF membranes were washed three times with TBST, and transferred to horseradish peroxidase-labeled goat anti-rabbit or anti-mouse secondary antibodies at room temperature for 1 h. Finally, PVDF membranes were washed with TBST three times and exposed to the luminescence reagent ECL. Images were captured with an image digital imaging system (General Electric Company, USA).

### Animals and experimental design

Four-week-old wild-type C57BL/6J male mice and db/db male mice (C57BL/6 J genetic background), were obtained from SLAC Laboratory Animal Co. Ltd. All mice were housed in a specific pathogen-free (SPF) barrier facility. The criteria for housing conditions were controlled temperature (23 °C ± 3 °C), 12 h light/dark cycle, standard chow feeding (Table S1) and free access to water.

The animals were randomized divided into three groups based on the gavage solution, including: (a) negative control group, C57BL/6J mice with water (*n* = 6); (b) positive control group, db/db mice with water (*n* = 6); (c) intervention group, db/db mice with C3G (*n* = 6). Professional technicians were employed to conduct the daily gavage with C3G aqueous solution (150 mg/kg/day) or equivalent volume of vehicle (pure water) for 6 weeks by using a soft, flexible and properly sized gavage needle. All procedures were strictly followed to prevent esophageal irritation or puncture, ensuring they were performed slowly and gently. Meanwhile, health monitoring of daily visual observations, behavioral evaluation, and weight measurements were included. A glucose monitor (Sinocare, China) was used to measure the fasting blood glucose of all mice.

All our experiments involving laboratory animals were conducted in strict adherence to the principles set forth by the National Institutes of Health guidelines on the care and use of laboratory animals. Additionally, the Animal Ethics Committee of Zhejiang Chinese Medical University granted approval for these procedures, dedicated by the authorization number 20201103-08.

### OGTT and HOMA-IR analysis

For oral glucose tolerance test (OGTT), mice were fasted for 12 h before experiment and glucose was administered orally (1.5 g/kg body weight) at 0 min. Blood glucose was measured at 0, 30, 60, 90, 120 and 150 min with a blood glucose monitor from a tail prick.

For homeostasis model assessment-insulin resistance (HOMA-IR) analysis, whole blood from the mice was centrifuged for 15 min at 4 °C and 4000 rpm to separate serum and plasma. The serum was collected and stored in a −80 °C freezer for later use. The levels of GLP-1 and insulin in mouse serum were determined using an ELISA kit according to the manufacturer’s instructions. HOMA-IR = serum glucose (mmol/L) × serum insulin (mU)/22.5.

### Statistical analyses

Fluorescent images and intensities of protein bands were counted by applying Image-Pro Plus-6.0 software and Image J software. SPSS 16.0 software was used to perform statistical analysis of data. Data were expressed as means ± standard deviation (SD). Statistical analyses for significance were performed using one-way ANOVA, Duncan’s multiple range test, and t-test, results were considered statistically significant if *p* < 0.05.

## Results

### Components of red bayberry anthocyanin extracts

**Fig. 1:**
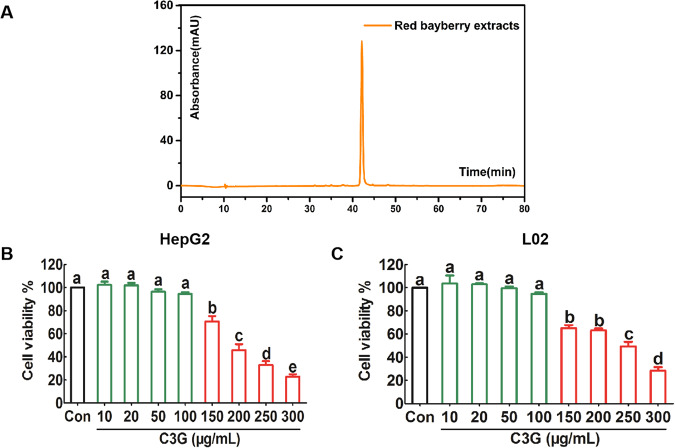
Components of anthocyanin extracted from red bayberry and its safe concentration for liver cells. HepG2 cells and L02 cells were incubated with different concentrations of C3G for 24 h. **A** Components of red bayberry extract were analyzed by UPLC. **B** The viability of HepG2 cells was analyzed by MTT assay. **C** The viability of L02 cells was analyzed by MTT assay. Any two histograms marked with different letters (a, b or c, etc.) are significantly different (*p* < 0.05).

Based on the UPLC analysis (at 520 nm), we inferred that the main component of anthocyanin extracts in red bayberry was C3G (Fig. 1A). Then, we tested the safe concentration range of C3G on hepatocytes by MTT assay. Our results showed that it is safe for HepG2 cells and L02 cells when the exposure concentration of C3G was lower than 100 µg/mL (Fig. 1B, C).

### C3G regulates glucose metabolism in liver cells with insulin resistance

**Fig. 2:**
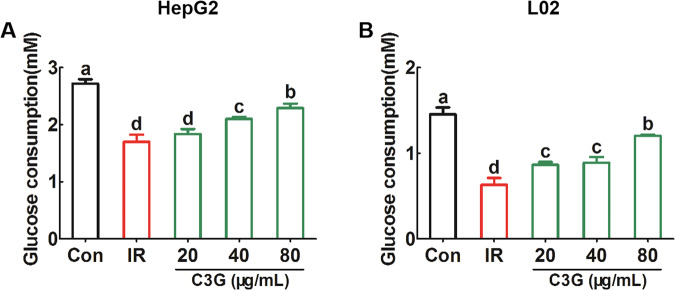
C3G restores reduced glucose consumption in IR hepatocytes. HepG2 and L02 cells were exposed to 0.2 mM PA and 30 mM glucose for 24 h, and continued to be incubated with 20, 40 and 80 μg/mL of C3G for another 24 h. **A** Glucose consumption of HepG2 cells after 24 h treatment. **B** Glucose consumption of L02 cells after 24 h treatment. Any two histograms marked with different letters (a, b or c, etc.) are significantly different (*p* < 0.05).

**Fig. 3:**
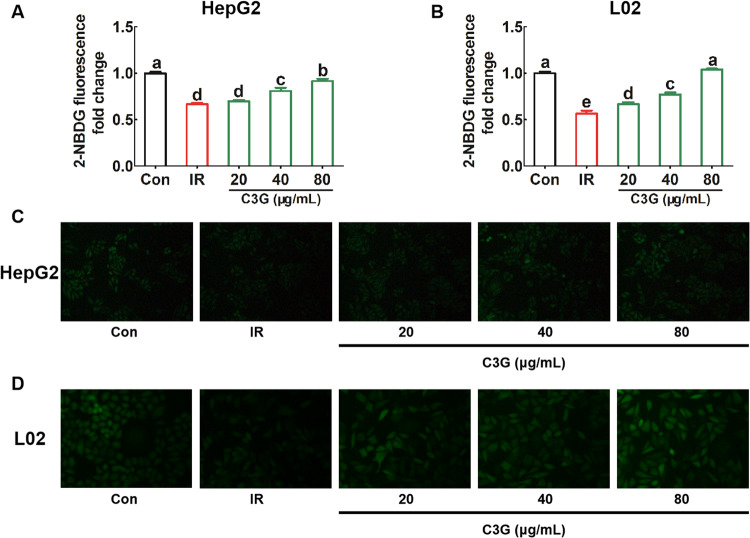
C3G restores reduced glucose uptake in IR hepatocytes. HepG2 and L02 cells were exposed to 0.2 mM PA and 30 mM glucose for 24 h, and continued to be incubated with 20, 40 and 80 μg/mL of C3G for another 24 h. **A** The quantitative analysis of 2-NBDG fluorescence in HepG2 cells. **B** The quantitative analysis of 2-NBDG fluorescence in L02 cells. **C** Uptake of 2-NBDG into HepG2 cells. **D** Uptake of 2-NBDG into L02 cells. Densitometry analysis was performed with Image-pro plus 6.0 software. Any two histograms marked with different letters (a, b or c, etc.) are significantly different (*p* < 0.05).

**Fig. 4:**
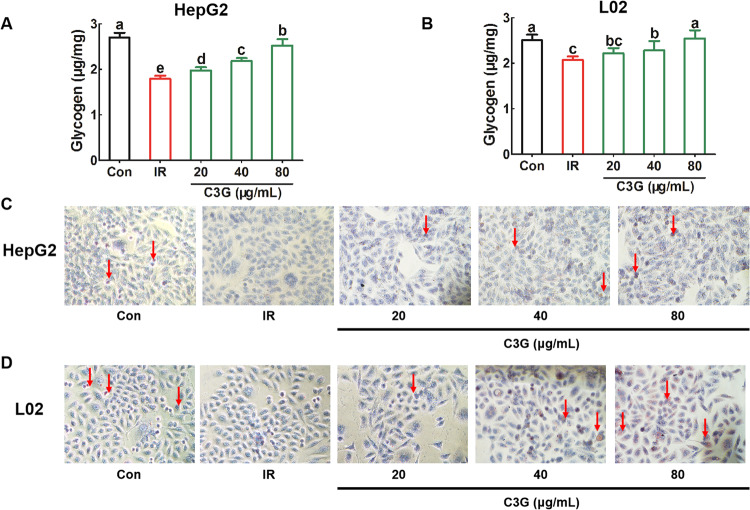
C3G increases glycogen synthesis in IR hepatocytes. HepG2 and L02 cells were exposed to 0.2 mM PA and 30 mM glucose for 24 h, and continued to be incubated with 20, 40 and 80 μg/mL of C3G for another 24 h. **A** Glycogen content in HepG2 cells. **B** Glycogen content in L02 cells. **C** Glycogen staining in HepG2 cells. **D** Glycogen staining in L02 cells. Any two histograms marked with different letters (a, b or c, etc.) are significantly different (*p* < 0.05).

**Fig. 5:**
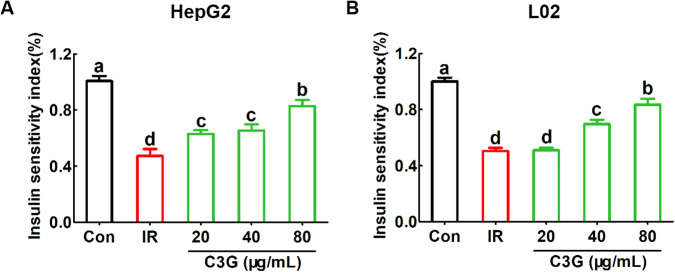
C3G enhances the insulin sensitivity of IR hepatocytes. HepG2 and L02 cells were exposed to 0.2 mM PA and 30 mM glucose for 24 h, and continued to be incubated with 20, 40 and 80 μg/mL of C3G for another 24 h. **A** Insulin sensitivity index of HepG2 cells. **B** Insulin sensitivity index of L02 cells. Any two histograms marked with different letters (a, b, or c, etc.) are significantly different (*p* < 0.05).

After incubating with 0.2 mM PA and 30 mM glucose for 24 h, compared with the untreated Con group, the obvious decrease in glucose consumption (Fig. 2A, B), glucose uptake (Fig. 3A, D) as well as glycogen content (Fig. 4A–D) in hepatocytes were found, which indicates that IR models in both HepG2 and L02 cells were successfully established.Further analysis showed that, compared with the IR model, C3G treatment significantly increased the glucose consumption (Fig. 2A, B) as well as the glucose uptake (Fig. 3A, B). Moreover, C3G at concentrations of 20, 40, and 80 μg/mL was able to prevent the inhibited glycogen synthesis that occurred during IR (Fig. 4A, D), with results similar to those of glucose consumption and glucose uptake. Additionally, during IR, we found that along with disturbances in glucose metabolism, the sensitivity of hepatocytes to insulin was significantly reduced. Furthermore, C3G can effectively improve this adverse effect (Fig. 5A, B). These results demonstrated that C3G was effective in alleviating IR in hepatocytes caused by HG and PA treatment.

To investigate the underlying molecular mechanism of C3G to ameliorate IR in hepatocytes, C3G at concentrations of 20, 40 and 80 μg/mL were chosen for following experiments.

### C3G improves insulin resistance by regulating PTP1B and p-IRS-2

**Fig. 6:**
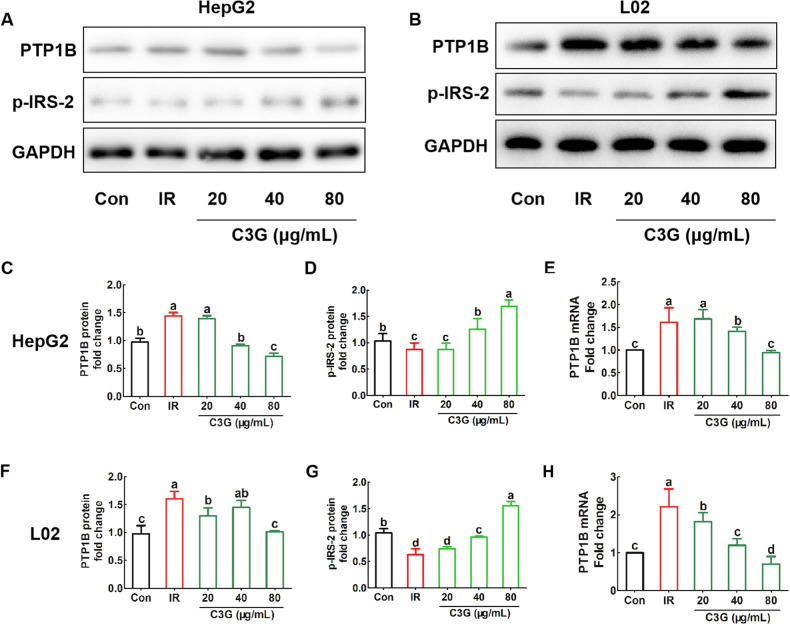
C3G alleviates insulin resistance via regulating PTP1B and p-IRS in hepatocytes. HepG2 and L02 cells were exposed to 0.2 mM PA and 30 mM glucose for 24 h, and continued to be incubated with 20, 40 and 80 μg/mL of C3G for another 24 h. **A** The protein bands of PTP1B and p-IRS in HepG2 cells. **B** The protein bands of PTP1B and p-IRS in L02 cells. **C**, **D** Bands of PTP1B and p-IRS were quantified and results were presented in a bar chart in HepG2 cells. GAPDH was used as a loading AQ6 control to ensure equal protein amounts. **E** The mRNA expression of PTP1B in HepG2 cells. **F**, **G** Bands of PTP1B and p-IRS were quantified and results were presented in a bar chart in L02 cells. GAPDH was used as a loading control to ensure equal protein amounts. **H** The mRNA expression of PTP1B in L02 cells. Any two histograms marked with different letters (a, b or c, etc.) are significantly different (*p* < 0.05).

Previous research has uncovered a significant increase in the PTP1B protein level in IR model cells induced by oleic acid. Moreover, when the PTP1B was inhibited, the IR of cells could be alleviated by increasing phosphorylation of insulin receptor substrate [17]. In Fig. 6, the mRNA levels (Fig. 6E, H) and protein expression (Fig. 6A–C, F) of PTP1B were both increased in our IR model cells, but it was restricted when cells were treated with C3G in a dose-dependent manner. Additionally, increased PTP1B can also produce a significant increase in p-IRS-2 (Tyr 612) in comparison with IR model cells (Fig. 6A, B, D, G), which may contribute to the improvement of insulin sensitivity in liver cells.

### C3G improves abnormal glucose tolerance and insulin resistance in diabetic db/db mice

**Fig. 7:**
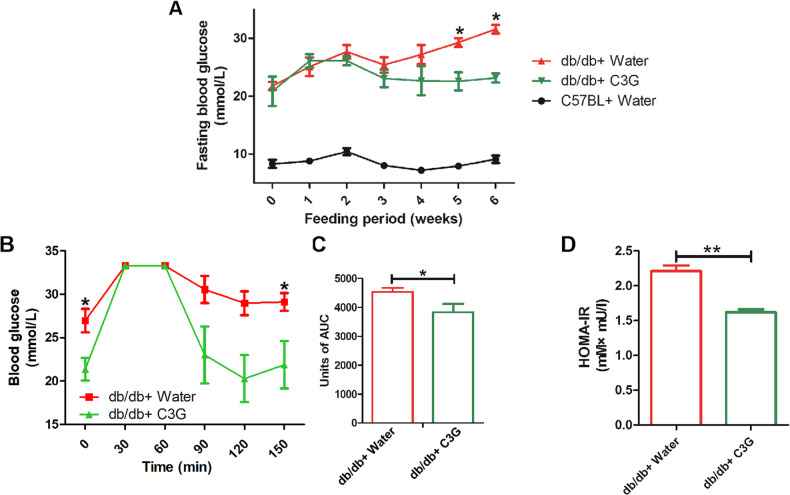
C3G can decrease fasting blood glucose and improve insulin resistance of db/db mice. Mice were gavaged with C3G aqueous solution or pure water for 6 weeks. **A** Fasting blood glucose during 6 weeks. **B**, **C** OGTT and AUC of db/db mice at 6-week. **D** HOMA-IR of db/db mice at 6-week. *p* < 0.05 was considered statistically significant, and *p < 0.05, **p < 0.01.

To verify the improvement of C3G on IR in vivo, we investigated the impact of C3G on diabetic db/db mice. At week 0, db/db mice (both positive control group and intervention group) exhibited abnormal hyperglycemic symptoms when compared to the C57BL/6J wild-type mice (negative control group), and there was no significant difference in blood glucose levels between two groups of db/db mice. However, supplementation with C3G for 6 weeks in the intervention group resulted in significantly reduced blood glucose levels compared to the positive control group (Fig. 7A).

Additionally, OGTT and HOMA-IR analyses analysis were performed to determine the effects of C3G intervention on glucose tolerance and IR. The blood glucose levels in db/db mice of the intervention group peaked at 30 min after injection, and recovered to levels close to baseline at 150 min (Fig. 7B). The area under curve (AUC) for blood glucose levels of the C3G-intervention group was significantly lower than that of the control group (Fig. 7C). However, no significant changes in blood glucose level in db/db mice of the positive control group was observed. Similarly, intervention group also showed a lower HOMA-IR score than the positive control group (Fig. 7D). These results provided an additional evidence for effectiveness of C3G in improving IR.

## Discussion

The findings of this study provide evidence to support our hypothesis that C3G has positive effects on glucose metabolism disorders associated with IR. Several lines of evidence demonstrated that IR plays a vital role in the development of T2DM [18–20], therefore, how to solve glucose metabolism disorders caused by IR may be a target in the treatment of this metabolic disease. Several studies have reported that anthocyanins can improve IR and hyperlipidemia in some rodent models of obesity and diabetes [21].

Compared to the normal cell, a cell with IR is a suitable in vitro model for investigating T2DM due to many glucose metabolism parameters could be altered. Hepatic cells are the most important cells in regulating glucose metabolism and are also the main target cells of insulin in vivo, playing a vital role in the pathogenesis of T2DM. Therefore, we aimed to further understand the beneficial effect of C3G on IR hepatocytes. In this study, to ensure broader applicability of the observed effects to liver cells, two distinct liver cell lines, HepG2 and L02, were used to eliminate cell line-specific variations. Firstly, 0.2 mM PA and 30 mM glucose were used to construct an IR model in HepG2 and L02 cells, which induced a decrease in glucose consumption, glucose uptake as well as glycogen content in both cell models. However, C3G treatment showed a significant increase in glucose consumption, glucose uptake as well as glycogen content in model cells (Figs. 2–4**)**.

Aside from hyperglycemia, the insufficient response to insulin by its target organs, is also a significant pathological condition associated with IR [22]. Our results clearly showed that C3G can significantly enhance the sensitivity of hepatocytes to insulin under IR condition (Fig. 5). The aforementioned data suggest that C3G could effectively ameliorate IR in hepatocytes.

Next, we further studied the molecular mechanism involved in the improvement of IR by C3G. Multiple signaling molecules are involved in the intracellular conduction and action of insulin, and extensive research has consistently demonstrated the significant role of IRS-2 in this process. For example, White et al. found that IRS-2-deficient mice exhibited diabetes due to a combination of IR and impaired insulin production [23]. Considering the phenotypes observed in insulin receptor-, IGF-1 receptor-, IRS-1-, and IRS-2-deficient mice [24–26], it can be concluded that IRS-2 functions as an insulin receptor substrate. Moreover, alternative studies have provided support for a model suggesting that the specificity of insulin sensitivity in hepatocytes is closely related to the formation of signaling complex involving insulin receptors and IRS-2 [27, 28]. Here, we have demonstrated that C3G increases the phosphorylation of IRS-2 in response to the IR model cells (Fig. 6).

In order to delve deeper into the role played by C3G in enhancing insulin signaling conducting, we noted that the role of PTP1B in negatively regulating insulin receptor kinase at the molecular level has established by Myers et al. [28], and Mice with the PTP1B gene removed exhibit characteristics that imply targeting PTP1B inhibition might be a promising strategy for treating diabetes [29]. Interestingly, the process of IRS-2 phosphorylation is blocked by PTP1B [30]. In our research, C3G was found to inhibit PTP1B expression, resulting in a direct increase in the phosphorylation of IRS-2 (Fig. 6). This phenomenon is consistent with the previous studies.

To further confirm the efficacy of C3G on IR, we have also performed in vivo experiments using db/db mice. The db/db mice have a deletion mutation in the leptin receptor (LepRdb/db) that results in abnormal splicing and a defective receptor for the adipocyte-derived hormone leptin [31], which leads to typical T2DM symptoms including abnormal hyperglycemia and IR in this mouse class [32], making it the most widely used model of T2DM. The dosage selection of C3G in vitro was based on prior cell toxicity and dose-response studies, as cellular response can be very sensitive to compound concentrations and can vary significantly from in vivo effects. Given the inherent differences between cellular and whole organism systems, it is not always feasible to maintain the exact dosage correlation between the two models. Therefore, the doses of C3G used for in vivo studies were defined in accordance with similar studies. In a previous study, db/db mice were administered 125 mg/kg body weight of mulberry anthocyanin, which contained approximately 47.2% C3G, via gavage to alleviate IR [11]. Furthermore, in another study, KK-Ay mice with glucose metabolism disorders were treated with 200 mg/kg Chinese red bayberry anthocyanin, which contained about 46.8% C3G [33]. Taking these two studies into consideration, we decided to set the dosage of C3G at 150 mg/kg body weight for mice. After a 6-week intervention with 150 mg/kg of C3G in db/db mice, an OGTT was performed. We found that the initial fasting blood glucose of mice in the C3G intervention group was significantly lower than that of control mice, while glucose intolerance of mice in the C3G intervention group was also significantly improved (Fig. 7B–D), and these results provide an additional evidence for the ameliorative effects of C3G in improving IR in vivo.

Several limitations are associated with this study. Regarding the result from OGTT, the omission of the control group (C57BL/6 J mice) in Fig. 7B–D limits our ability to draw definitive conclusions about the degree of glucose intolerance in the db/db+water group. Therefore, we plan to incorporate pairwise comparisons between the db/db+water group and the C57BL/6J control group to quantify the extent of glucose intolerance and IR manifested by the db/db mice. In addition, in our in vivo experiment, we performed OGTT and calculated the HOMA-IR indices in diabetic db/db mice. However, we acknowledge that these results alone do not constitute a robust demonstration that C3G significantly improves IR in this model. To solidify our findings, we propose additional future experiments that include conducting insulin tolerance test, utilizing hyperinsulinemic-euglycemic clamp techniques to provide a direct measure of insulin sensitivity, and quantifying molecular markers associated with IR pathways. Combining these comprehensive measures to substantiate the ameliorative effects of C3G on IR in the db/db mouse model.

## Conclusions

In summary, we demonstrated the beneficial effects of anthocyanin C3G on glucose metabolic disorders due to IR in subjects with T2DM. In vitro, the protective mechanism by C3G in hepatocyte with IR was closely correlated to the increase in glucose consumption and glycogen synthesis, as well as the enhancement of insulin sensitivity, which involves the regulation of PTP1B and p-IRS-2. Furthermore, it was observed that intervention with C3G ameliorated glucose tolerance and mitigated IR to a certain extent in diabetic db/db mice. Therefore, anthocyanin C3G as a dietary supplementation has the potential to reverse the glucose metabolic aberrations that are associated with IR during T2DM. This research offers additional insight into using natural products for the treatment of chronic diseases.

### Supplementary information

Supplementary Information

## Data Availability

The data that support the findings of this study are available from the corresponding author upon reasonable request.
