# Supplementary material for: Anti-diabetic effect of anthocyanin cyanidin-3-O-glucoside: data from insulin resistant hepatocyte and diabetic mouse
Source: Nutr Diabetes. 2024 Mar 1;14:7. doi: 10.1038/s41387-024-00265-7 (PMC10907696; doi:10.1038/s41387-024-00265-7)
Supplement: Supplementary file 1 — Supplementary Information [file 41387_2024_265_MOESM1_ESM.doc]

**Table S1** The base composition of diet fed to mice.

| Component | Content | Component | Content |
| --- | --- | --- | --- |
| Moisture (%) | 8.6 | Calcium (%) | 1.24 |
| Crude protein (%) | 18.8 | Phosphorus (%) | 0.83 |
| Crude fat (%) | 16.2 | Calcium:Phosphorus | 1.49 |
| Crude ash (%) | 5.2 | Lysine (%) | 1.38 |
| Crude fibre (%) | 3.98 | Methionine + Cystine (%) | 0.78 |
| Nitrogen-free leachate (%) | 45.2 | Energy (kcal) | 379 |
